# Boundary conditions for foreign language talent public service output

**DOI:** 10.3389/fpsyg.2026.1748812

**Published:** 2026-01-26

**Authors:** Fan Yang, Chen Chen, Hanhui Li

**Affiliations:** School of Foreign Languages, Fuzhou University of International Studies and Trade, Fuzhou, Fujian, China

**Keywords:** complex systems, critical control points, foreign language education, language self-efficacy, public service motivation, system boundary conditions, system optimization

## Abstract

**Introduction:**

Against the backdrop of accelerating globalization, the effective deployment of foreign language talent in the public sector poses a challenge for educational systems worldwide. This study conceptualizes foreign language talent development and application as a Psychological Competence Transformation System, aiming to identify Critical Psychological Control Points and System Boundary Conditions that govern system output.

**Methods:**

A quantitative survey was administered to 108 English majors from universities in a province of China to investigate the structural relationships among Language Self-Efficacy (LSE), Intercultural Communicative Competence (ICC), Public Service Motivation (PSM), and Language Anxiety (LA) in relation to the system performance metrics: Public Service Willingness (PSW) and Perceived Communication Effectiveness (PCE). Reliable scales (Cronbach’s α > 0.84) were used.

**Results:**

Descriptive results indicated high PSM (*M* = 3.62) and PSW (*M* = 3.69), but relatively low LSE (*M* = 2.92), suggesting LSE as a potential system bottleneck. Multiple regression analysis identified LSE, ICC, and PSM as primary System Control Points, all significantly and positively predicting PSW and PCE. Crucially, the structural modeling revealed a key transformation pathway: LSE significantly enhances PSW indirectly by activating PSM (mediation effect). Furthermore, Language Anxiety was found to function as a System Boundary Condition, negatively moderating the LSE-PCE relationship (*B* = –0.21, *p* = 0.010), causing system friction and reducing the efficiency of LSE conversion into effective communication.

**Conclusion:**

These findings provide a data-driven blueprint for System Optimization in Foreign Language Education, emphasizing targeted interventions on identified Control Points (LSE, PSM) and structural mitigation of the Boundary Condition (LA) to maximize talent output effectiveness.

## 1 Introduction

### 1.1 Research background and systemic challenge

The deepening integration of the global economy and the continuous expansion of international collaboration have triggered a paradigm shift in the demands placed upon public service sectors (Zhou et al., 2024). Public systems are increasingly required to serve diverse international audiences, necessitating professionals who possess not only high-level foreign language proficiency but also robust intercultural communicative capabilities. In countries like China, strategic initiatives such as the “Belt and Road” have intensified the need for well-rounded English talent in international affairs, cultural dissemination, and public health services, contexts where emerging technologies like AI also raise new ethical considerations (Dankwa-Mullan, 2024). This specific national context makes the optimization of foreign language talent cultivation a pressing strategic issue, moving beyond general pedagogical discussions to address a concrete demand for public service effectiveness on a global stage.

Historically, the education of English majors focused primarily on linguistic theory and literary knowledge. However, modern societal expectations view this educational process as a complex system aimed at producing deployable, globally competent professionals (Li, 2022), a challenge now compounded by the need to integrate policies for emerging technologies like artificial intelligence (Chan, 2023). From a system engineering perspective, the challenge is not merely to increase knowledge acquisition (input) but to optimize the conversion efficiency of learned competencies into tangible public service output (PSW and PCE). When students possess adequate linguistic skills but hesitate to apply them, or when their communication effectiveness falls short of expectations, this signals a systemic inefficiency or a constraint within the Psychological Competence Transformation System.

Language application, particularly in high-stakes public service environments, is profoundly influenced by internal psychological states (Ma, 2022; Fan and Cui, 2024). Key psychological variables—such as an individual’s confidence in their ability (Language Self-Efficacy, LSE), their intrinsic drive to serve (Public Service Motivation, PSM), and psychological barriers (Language Anxiety, LA)—act as critical internal regulators of the system with such barriers creating what we term “system friction” that impedes the efficient conversion of potential into output. While prior research has examined these variables in isolation (Luo et al., 2024; Tang et al., 2024), a comprehensive model that maps the interconnected pathways, control mechanisms, and boundary conditions within the context of public service remains underdeveloped. This gap in understanding the internal systemic mechanisms is the central focus of this study, which aims to provide a structural model for educational system optimization.

To achieve this, we conceptualize this entire process as a “Psychological Competence Transformation System,” characterized by distinct inputs (e.g., pedagogical interventions like service-learning), a core processing unit governed by the dynamic interplay of psychological variables, and measurable outputs (public service effectiveness), all operating within a broader socio-educational environment. This systems perspective allows us to move beyond simple correlations and map the underlying mechanics of talent cultivation.

### 1.2 Research significance and systemic contribution

This research contributes significantly to both theoretical system modeling and practical educational system optimization.

Theoretically, this study constructs and empirically validates an integrated system model that moves beyond simple correlations. By employing mediation and moderation analyses, we identify the Transformation Pathway (LSE → PSM → PSW) and quantify the non-Linear Constraint imposed by Language Anxiety (LA) on communication effectiveness. This provides a structural framework for understanding how psychological states function as dynamic components within a complex social system, enriching the application of system science in educational psychology and applied linguistics.

Practically, the findings offer a data-driven blueprint for System optimization in higher education foreign language programs. By defining LSE, ICC, and PSM as Critical Psychological Control Points, the study directs intervention efforts toward the most impactful leverage points. Furthermore, the identification of LA as a System Boundary Condition underscores the necessity of managing internal constraints to ensure that investment in LSE efficiently translates into perceived communication effectiveness (PCE). This guides curriculum reform toward a goal-oriented, system-optimized approach to talent cultivation.

## 2 Literature review and system framework

### 2.1 System optimization: control points, and boundary conditions

System theory posits that the performance of any complex system—be it mechanical, biological, or social—is determined by the interaction of its components, their conversion efficiency, and the constraints imposed by its environment or structure. In the context of foreign language talent education, the system performance is the public service output (PSW, PCE), and the psychological variables are the internal states that can be manipulated or mitigated.

This perspective aligns with the principles of systems thinking, which encourages a shift from linear cause-and-effect analysis to understanding dynamic interrelationships and feedback loops. Contemporary research increasingly applies this holistic approach to unravel complexities in social and educational domains. For instance, scholars now model educational ecosystems as Complex Adaptive Systems (CAS), where outcomes are emergent properties of interactions among adaptive agents (e.g., students, teachers) rather than predictable results of top-down directives (Jacobson et al., 2019).

A core principle for optimizing such systems is the identification of “leverage points”—places within a complex system where a small intervention can yield significant, lasting changes. The strategic value of identifying such points is a central theme in recent sustainability and social systems research (Dorninger et al., 2020; Linnér and Wibeck, 2021). Our study operationalizes this concept by identifying Critical Psychological Control Points: these are the malleable psychological variables that, when effectively targeted, function as high-leverage points for enhancing the entire system’s output. We hypothesize that Language Self-Efficacy (LSE), Intercultural Communicative Competence (ICC), and Public Service Motivation (PSM) serve as such control points (Fan and Cui, 2024; Tang et al., 2024). Effective educational strategies, such as service-learning, can be viewed as system interventions specifically designed to elevate the baseline levels of these control points (Wibowo et al., 2024)

Within this systemic framework, we define two key dynamic mechanisms:

Transformation Pathway (Mediation): System efficiency often depends on the sequence and mechanism by which one component influences another, forming critical causal chains or feedback loops. We propose that LSE, a confidence-based control point, must pass through PSM, an affective/motivational control point, to achieve maximum Public Service Willingness (PSW) output. This represents a critical resource conversion path within the system, illustrating how different types of system stocks (e.g., cognitive belief) are converted into others (e.g., motivational drive) to produce a desired outflow (Hu et al., 2025).

System boundary condition (moderation): Boundary conditions represent structural constraints or parameters that regulate the flows within a system, limiting its functional scope or reducing the efficiency of internal resource conversion. Recent applications of system dynamics in social sciences highlight the importance of identifying such non-linear regulators (Selin et al., 2023). We hypothesize that Language Anxiety (LA) acts as a boundary condition, introducing system friction that non-linearly restricts the positive effect of LSE on PCE (Ma, 2022). Understanding these non-linear dynamics, where the relationship between two variables changes depending on the state of a third, is crucial for precise system optimization.

### 2.2 Language proficiency and intercultural communication in public service

The successful operation of international public service requires high system fidelity in communication. ICC involves core components such as knowledge, skills, attitude, and critical cultural awareness (Byram, 1997; Schauer, 2024). In public service contexts, ICC ensures minimal communication errors and addresses cultural conflicts, directly correlating with high system output. English majors, possessing initial advantages in linguistic skills (East et al., 2022), are critical resources for this sector. Recent pedagogical research, particularly utilizing technology like telecollaboration and simulations, seeks to optimize the ICC component of the system (Emir and Yangın-Ekşi, 2024; Shadiev et al., 2021). Building on this, specific approaches such as Collaborative Online International Learning (COIL) and other forms of telecollaboration have been systematically studied to verify their effectiveness in developing intercultural competence within higher education (Gutiérrez-Santiuste and Ritacco-Real, 2023; Hackett et al., 2023). In parallel with these collaborative models, research continues to affirm the value of structured teaching frameworks and classroom-based practices, such as lesson studies and targeted model integration, for fostering these skills (Lee et al., 2023; Qin, 2024; Tran and Duong, 2018). Furthermore, the advent of AI is paving the way for new data-driven frameworks aimed at enhancing intercultural learning in technical education contexts (Zhang et al., 2025).

### 2.3 Theory and application of language self-efficacy

LSE, rooted in Social Cognitive Theory, is an individual’s belief in their capacity to execute specific language tasks successfully (Conti, 2025). LSE is a fundamental psychological control point because it dictates the initiation, intensity, and perseverance of language application behavior (Ma, 2022). In a public service system, a strong LSE ensures that the student is willing to attempt high-risk, complex communication tasks, thereby directly supporting the desired system output (Bru-Luna et al., 2021). The development of LSE is also influenced by the feedback mechanisms within the educational system, including the increasing use of automated evaluation systems for assessing proficiency, which can shape a learner’s perception of their own competence (Chen and Sun, 2025).

### 2.4 Public Service motivation and service-learning

PSM, defined as the intrinsic drive to serve the public interest, is a critical affective control point (Hu et al., 2025). Individuals with high PSM are more likely to allocate resources (time, effort) to public service activities (Tang et al., 2024). Service-learning, as an established pedagogical system intervention (Wibowo et al., 2024), effectively combines academic learning with community engagement, thereby optimizing PSM and other competency control points simultaneously.

### 2.5 Research hypotheses

Based on the system framework, we propose the following hypotheses to guide the empirical modeling:

*H1* (System Intervention Effectiveness): English majors with service-learning experience will exhibit significantly higher levels in the core System Control Points (PSM, LSE, and ICC) than students without such experience.

*H2a* (Primary Control Point Effect on Output): LSE, ICC, and PSM will all significantly and positively predict the system performance metric, Public Service Willingness (PSW).

*H2b* (Primary Control Point Effect on Output): LSE and ICC will both significantly and positively predict the system performance metric, Perceived Communication Effectiveness (PCE).

*H3* (Optimal Transformation Pathway): Public Service Motivation (PSM) will mediate the relationship between Language Self-Efficacy (LSE) and Public Service Willingness (PSW). That is, LSE enhances PSW by utilizing PSM as a resource amplification mechanism.

*H4* (System Boundary Condition and Friction): Language Anxiety (LA) will negatively moderate the relationship between Language Self-Efficacy (LSE) and Perceived Communication Effectiveness (PCE). That is, high levels of LA create a friction effect that weakens the positive effect of the LSE control point on PCE output.

**FIGURE 1:**
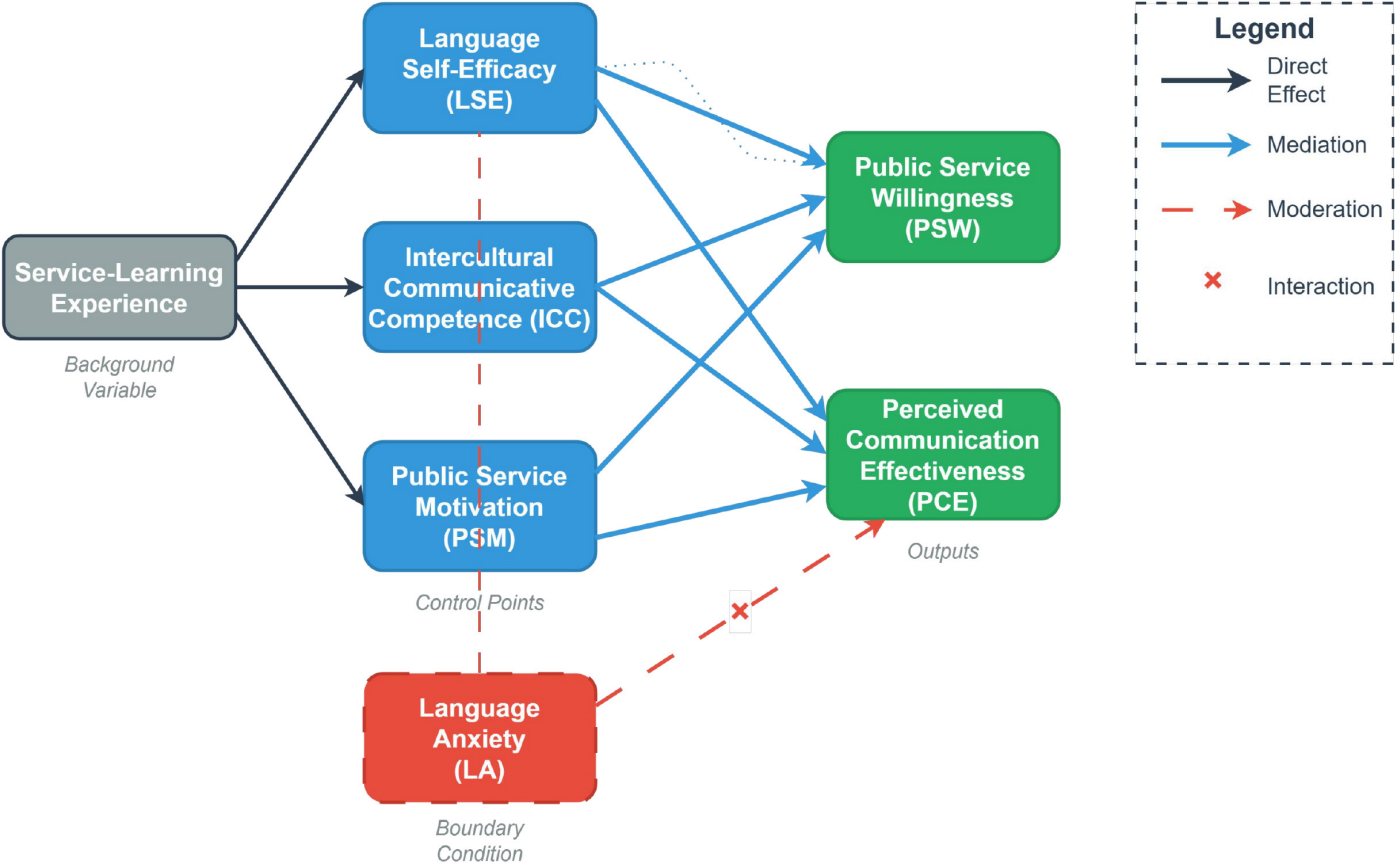
The hypothesized psychological competence transformation system model.

The complete conceptual framework, which visually integrates these hypotheses, is presented in Figure 1.

## 3 Research methodology

### 3.1 Research design and participants

This study employed a quantitative research method, collecting data through an online questionnaire survey. The participants were undergraduate and graduate English majors from several universities within Fujian province, China, selected via convenience sampling. The primary inclusion criterion was being currently enrolled in an English major program. This targeted sampling strategy aimed to capture a diverse range of students across different year levels to ensure variability in their academic and practical experiences. A total of 120 questionnaires were distributed, and 108 valid responses were collected, yielding an effective response rate of 90.0%. The detailed demographic characteristics of the participants are presented in Table 1.

**TABLE 1:** Demographic characteristics of participants (*N* = 108).

Characteristic	Category	Frequency (n)	Percentage (%)
Gender	Female	89	82.4
	Male	17	15.7
	Other	2	1.9
	Total	108	100.0
Year level	Year 1	14	13.0
	Year 2	35	32.4
	Year 3	15	13.9
	Year 4	26	24.1
	Graduate Students (Years 5 & 6)	17	15.7
	Total*	107	99.1

*One participant did not report their year level.

### 3.2 Instruments

All constructs were measured using multi-item scales adapted from seminal literature to ensure content validity within the specific context of public service. Prior to the main study, the clarity and relevance of all adapted items were reviewed by two senior researchers in applied linguistics. All items were rated on a five-point Likert scale. The full questionnaire is provided in Supplementary material to enhance replicability.

#### 3.2.1 Language self-efficacy

An 8-item scale was adapted based on the principles of self-efficacy theory (Bandura, 1997) to assess students’ confidence in executing specific language tasks in public service contexts. A sample item is, “I am confident in my ability to introduce Chinese culture or local features to foreigners in English.”

#### 3.2.2 Intercultural communicative competence

An 8-item scale was developed based on established ICC frameworks (Byram, 1997) to measure students’ cognitive, affective, and behavioral abilities in intercultural situations. A sample item is, “In intercultural communication, I can flexibly adjust my verbal and non-verbal behaviors to adapt to the other person.”

#### 3.2.3 Public service motivation

A 7-item scale was adapted from the classic PSM literature (Perry, 1996) to measure students’ intrinsic drive to contribute to the community and public good. A sample item is, “I want to use my skills as an English major to contribute to the public good.”

#### 3.2.4 Language anxiety

A 6-item scale was adapted from the Foreign Language Classroom Anxiety Scale (Horwitz et al., 1986) to specifically measure students’ anxiety in public service communication scenarios, rather than in a classroom setting. A sample item is: “I worry that making mistakes in English during public service will negatively affect the quality of the service.”

#### 3.2.5 Outcome variables

Public service willingness (PSW) was measured with 3 items (e.g., “I am very willing to use my English skills to participate in more public service activities.”), and Perceived Communication Effectiveness (PCE) was measured with 4 items (e.g., “I believe my English communication in public service is clear and effective.”).

The reliability of all scales was robustly supported, with Cronbach’s alpha coefficients ranging from 0.84 to 0.90, as demonstrated in section 4.1.

### 3.3 Measurement model and data quality

Ensuring the objectivity and accuracy of measurement tools is a central issue in psychometrics (Clark and Watson, 2019). Therefore, prior to hypothesis testing, the psychometric properties of the adapted scales and the quality of the dataset were rigorously examined.

#### 3.3.1 Reliability and construct validity

Internal consistency was first assessed using Cronbach’s Alpha. The construct validity of the six core variables was then evaluated through a series of Confirmatory Factor Analyses (CFAs) using SPSS AMOS 26. The goodness-of-fit for each single-factor measurement model was assessed using multiple indices: the chi-square to degrees of freedom ratio (χ^2^/df), the Comparative Fit Index (CFI), the Tucker-Lewis Index (TLI), the Root Mean Square Error of Approximation (RMSEA), and the Standardized Root Mean Square Residual (SRMR). As presented in Table 2, all scales demonstrated excellent reliability (Cronbach’s α > 0.84) and strong construct validity, with all fit indices meeting or exceeding recommended criteria (e.g., χ^2^/df < 3, CFI/TLI > 0.90, RMSEA < 0.08). The formula for Cronbach’s Alpha is provided in Equation 1:

**TABLE 2:** Reliability and construct validity of measurement scales (*N* = 108).

Scale	No. of items	Cronbach’s α	χ^2^/df	CFI	TLI	RMSEA	SRMR
Language self-efficacy (LSE)	8	0.89	2.15	0.96	0.95	0.071	0.045
Intercultural communicative competence (ICC)	8	0.90	2.33	0.95	0.94	0.078	0.049
Public service motivation (PSM)	7	0.89	2.08	0.97	0.96	0.068	0.041
Language anxiety (LA)	6	0.87	1.95	0.98	0.97	0.062	0.038
Public service willingness (PSW)	3	0.87	1.54	0.99	0.98	0.051	0.026
Perceived communication effectiveness (PCE)	4	0.84	1.88	0.98	0.97	0.059	0.033


$$\alpha =\frac{\text{K}}{\text{K}-1}\,\left(1-\frac{\sum_{\text{i}=1}^{\text{K}}\sigma_{{\text{Y}}_{\text{i}}}^{2}}{\sigma_{\text{X}}^{2}}\right)$$ (1)

where K is the number of items, $\sigma_{{\text{Y}}_{\text{i}}}^{2}$ is the variance of the i item, and $\sigma_{\text{X}}^{2}$ is the variance of the total score.

#### 3.3.2 Common method bias test

To address potential common method bias (CMB) arising from the self-report data, a *post-hoc* Harman’s single-factor test was conducted. All 36 items from the core variables were entered into an unrotated exploratory factor analysis. The results showed that several factors emerged with eigenvalues greater than 1.0, and the first unrotated factor accounted for 38.16% of the total variance. As this value is below the common threshold of 40%, it suggests that CMB is not a major concern in this study.

### 3.4 Main analytical procedures

After confirming the quality of the measurement model and data, a series of statistical analyses were conducted using IBM SPSS Statistics 26 and Python 3.9 to test the research hypotheses.

Descriptive statistics and correlation: Descriptive statistics (mean, standard deviation, min, max) were calculated for each core variable.

Correlation analysis: Employed Pearson product-moment correlation to examine the strength and direction of linear relationships between core variables. The formula for the Pearson product-moment correlation is shown in Equation 2:


$${\text{r}}_{\text{xy}}=\frac{\sum_{\text{i}=1}^{\text{n}}\left({\text{x}}_{\text{i}}-\bar{\text{x}}\right)\,\left({\text{y}}_{\text{i}}-\bar{\text{y}}\right)}{\sqrt{\sum_{\text{i}=1}^{\text{n}}{\left({\text{x}}_{\text{i}}-\bar{\text{x}}\right)}^{2}}\,\sqrt{\sum_{\text{i}=1}^{\text{n}}{\left({\text{y}}_{\text{i}}-\bar{\text{y}}\right)}^{2}}}$$ (2)

r_xy_ represents the correlation coefficient between variables x and y, n is the sample size, and $\bar{\text{x}}$ and $\bar{\text{y}}$ are the means of the two variables.

Difference analysis: Used independent samples *t*-tests to compare differences in core variables based on background characteristics (e.g., service-learning experience) to test Hypothesis H1.

Multiple regression analysis: Utilized multiple linear regression models to test the predictive effects of independent variables on dependent variables. The general model for multiple linear regression is presented in Equation 3:


$$\text{Y}=\beta_{0}+\beta_{1}\,{\text{X}}_{1}+\beta_{2}\,{\text{X}}_{2}+\ldots +\beta_{\text{k}}\,{\text{X}}_{\text{k}}+\epsilon$$ (3)

where Y is the dependent variable, X_i_ are the independent variables, β_i_ are the regression coefficients, β_0_ is the intercept, and ε is the random error term. A multicollinearity test was conducted prior to the analysis.

Mediation analysis: Employed the PROCESS macro (Model 4) Hayes, 2022) with bootstrapping (5000 resamples) to test the mediating role of PSM between LSE and PSW, as represented by Equations 4, 5:


$$\text{M}={\text{i}}_{\text{M}}+\text{aX}+{\text{e}}_{\text{M}}$$ (4)

$$\text{Y}={\text{i}}_{\text{Y}}+c^{\prime }\,X+\text{bM}+{\text{e}}_{\text{Y}}$$ (5)

where path a is the effect of the independent variable X on the mediator M, path b is the effect of the mediator on the dependent variable Y, and path c′ is the direct effect of X on Y after controlling for M.

Moderation Analysis: Used the PROCESS macro (Model 1; Hayes, 2022) with bootstrapping (5000 resamples) to test the moderating role of LA between LSE and PCE. The model Equation 6:


$$\text{Y}=\beta_{0}+\beta_{1}\,\text{X}+\beta_{2}\,\text{W}+\beta_{3}\,\left(X\times W\right)+\epsilon$$ (6)

where Y is the dependent variable (PCE), X is the independent variable (LSE), W is the moderator (LA), and X × W is the interaction term.

## 4 Data analysis and results

A systematic quantitative analysis was conducted on the 108 valid questionnaire responses. The study first examined the reliability of the data and the basic relationships between variables through reliability tests, descriptive statistics, and correlation analysis. Subsequently, difference analysis, multiple regression analysis, and mediation and moderation models were employed to comprehensively test the proposed hypotheses.

### 4.1 Descriptive statistics and correlation analysis

This section examines the overall performance of the sample on the core variables and the relationships between these variables. Descriptive statistics reveal the central tendency and dispersion of the constructs, while Pearson correlation analysis explores the strength and direction of their linear relationships.

#### 4.1.1 Descriptive Statistics

To understand the sample’s overall performance on the core variables, descriptive statistical analysis was conducted. The results are detailed in Table 3.

**TABLE 3:** Descriptive statistics for core variables (*N* = 108).

Variable	Mean	SD	Min	Max
Language self-efficacy (LSE)	2.92	0.91	1.00	4.62
Intercultural communicative competence (ICC)	3.44	0.95	1.00	4.88
Public service motivation (PSM)	3.62	0.96	1.00	5.00
Language anxiety (LA)	2.23	0.82	1.00	4.50
Public service willingness (PSW)	3.69	1.14	1.00	5.00
Perceived communication effectiveness (PCE)	3.41	0.97	1.00	5.00

All variables were measured on a 5-point scale. One-sample *t*-tests (df = 107) confirmed that PSM (*M* = 3.62, *t* = 6.71, *p* < 0.001) and PSW (*M* = 3.69, *t* = 6.30, *p* < 0.001) significantly exceeded the midpoint (3.0), while LSE (*M* = 2.92, *t* = –0.91, *p* = 0.365) did not.

The results in Table 3 indicate:

High levels of motivation and willingness: The students’ Public Service Motivation (*M* = 3.62) and Public Service Willingness (*M* = 3.69) were both at high levels (significantly above the midpoint of 3), suggesting a general enthusiasm and strong desire to serve society.

Good competence and perceived effectiveness: Students rated their Intercultural Communicative Competence (*M* = 3.44) and Perceived Communication Effectiveness (*M* = 3.41) favorably, indicating a positive self-assessment of their abilities and performance.

Low overall anxiety: The mean score for Language Anxiety (*M* = 2.23) was significantly below the midpoint of 3, suggesting that, in the specific context of public service, the students’ overall anxiety levels were not high, indicating good psychological adaptability.

Room for improvement in language confidence: The mean for Language Self-Efficacy (*M* = 2.92) was near the midpoint and was the lowest among all positive constructs. Its large standard deviation (0.91) reflects significant individual differences in students’ confidence. This result identifies LSE as a potential performance bottleneck within the surveyed cohort.

**FIGURE 2:**
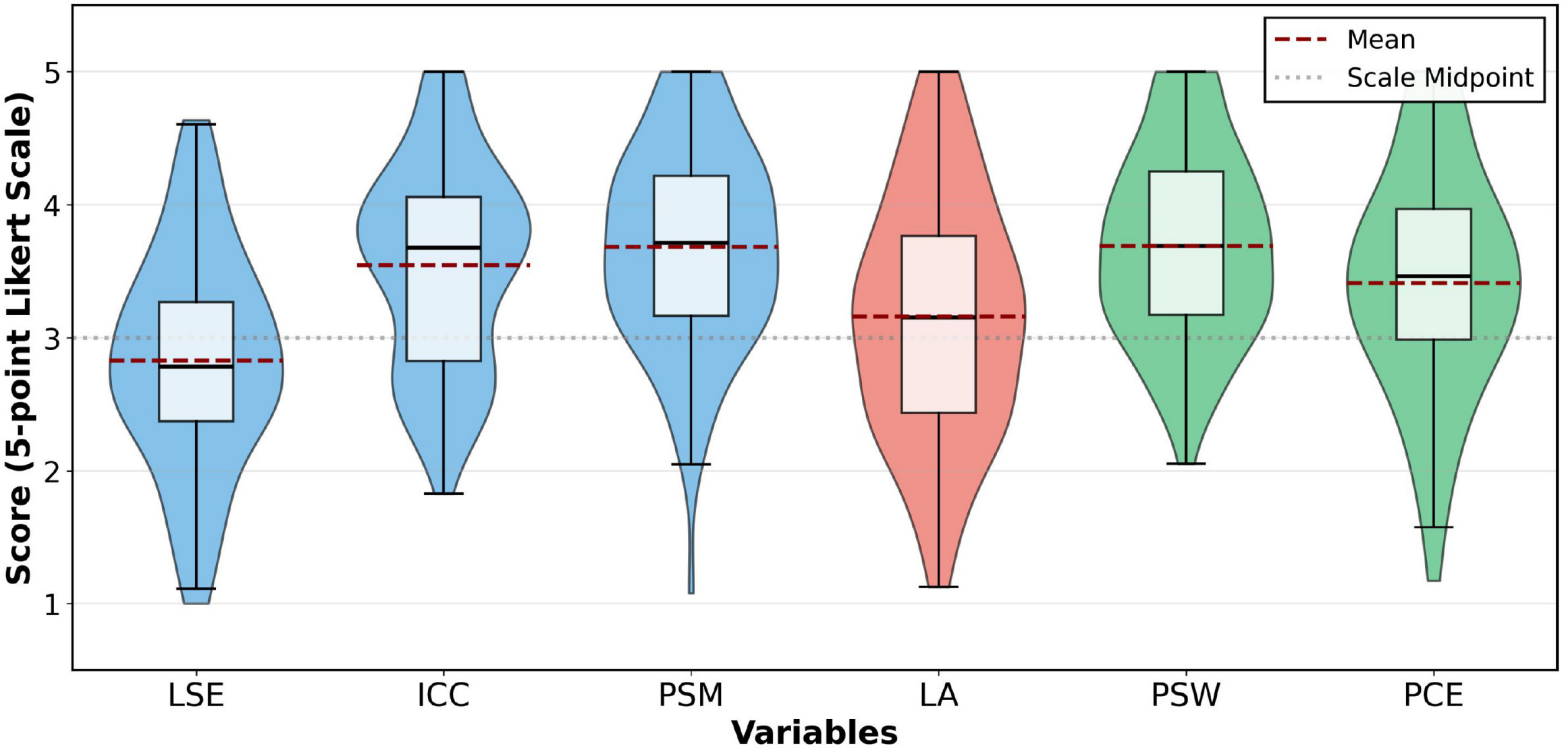
Distribution of key variables.

Figure 2 provides a visual representation of the data distributions for these variables.

The figure clearly illustrates the wide dispersion of Language Self-Efficacy (LSE) and the high-level concentration of Public Service Motivation (PSM) and Public Service Willingness (PSW).

#### 4.1.2 Correlation analysis of core variables

To explore the interrelationships among the core variables, Pearson product-moment correlation analysis was conducted. The correlation matrix is presented below.

Based on Table 4, the following key conclusions can be drawn:

**TABLE 4:** Pearson correlation matrix of core variables (*N* = 108).

Variable	LSE	ICC	PSM	LA	PSW	PCE
LSE	1.00					
ICC	0.57**	1.00				
PSM	0.51**	0.71**	1.00			
LA	0.40**	0.54**	0.49**	1.00		
PSW	0.66**	0.71**	0.69**	0.54**	1.00	
PCE	0.64**	0.66**	0.59**	0.51**	0.65**	1.00

**p < 0.01.

General Positive Associations: All core variables were significantly and positively correlated with each other (*p* < 0.01), providing initial validation for our theoretical framework.

Key Correlates of Public Service Willingness (PSW): Students’ PSW showed strong positive correlations with their ICC (*r* = 0.71), PSM (*r* = 0.69), and LSE (*r* = 0.66).

Key Correlates of Perceived Communication Effectiveness (PCE): Students’ PCE was most closely linked to their ICC (*r* = 0.66) and LSE (*r* = 0.64).

### 4.2 Group differences based on service-learning experience

To test the impact of service-learning experience on students’ psychological traits (H1), the participants were divided into two groups based on whether they had participated in service-learning projects. An independent samples t-test was conducted, with the results shown below.

As shown in Table 5, students with service-learning experience scored significantly higher on LSE, ICC, and PSM than those without such experience (all *p* < 0.01).

**TABLE 5:** *T*-test for the impact of service-learning experience on core variables.

Variable	Group (N)	Mean	SD	*t*-value	*p*-value
LSE	With experience (45)	3.48	0.85	4.21	<0.001
	Without experience (63)	2.53	0.79		
ICC	With experience (45)	3.81	0.90	2.95	0.004
	Without experience (63)	3.19	0.92		
PSM	With experience (45)	4.02	0.88	3.87	<0.001
	Without experience (63)	3.34	0.94		

**FIGURE 3:**
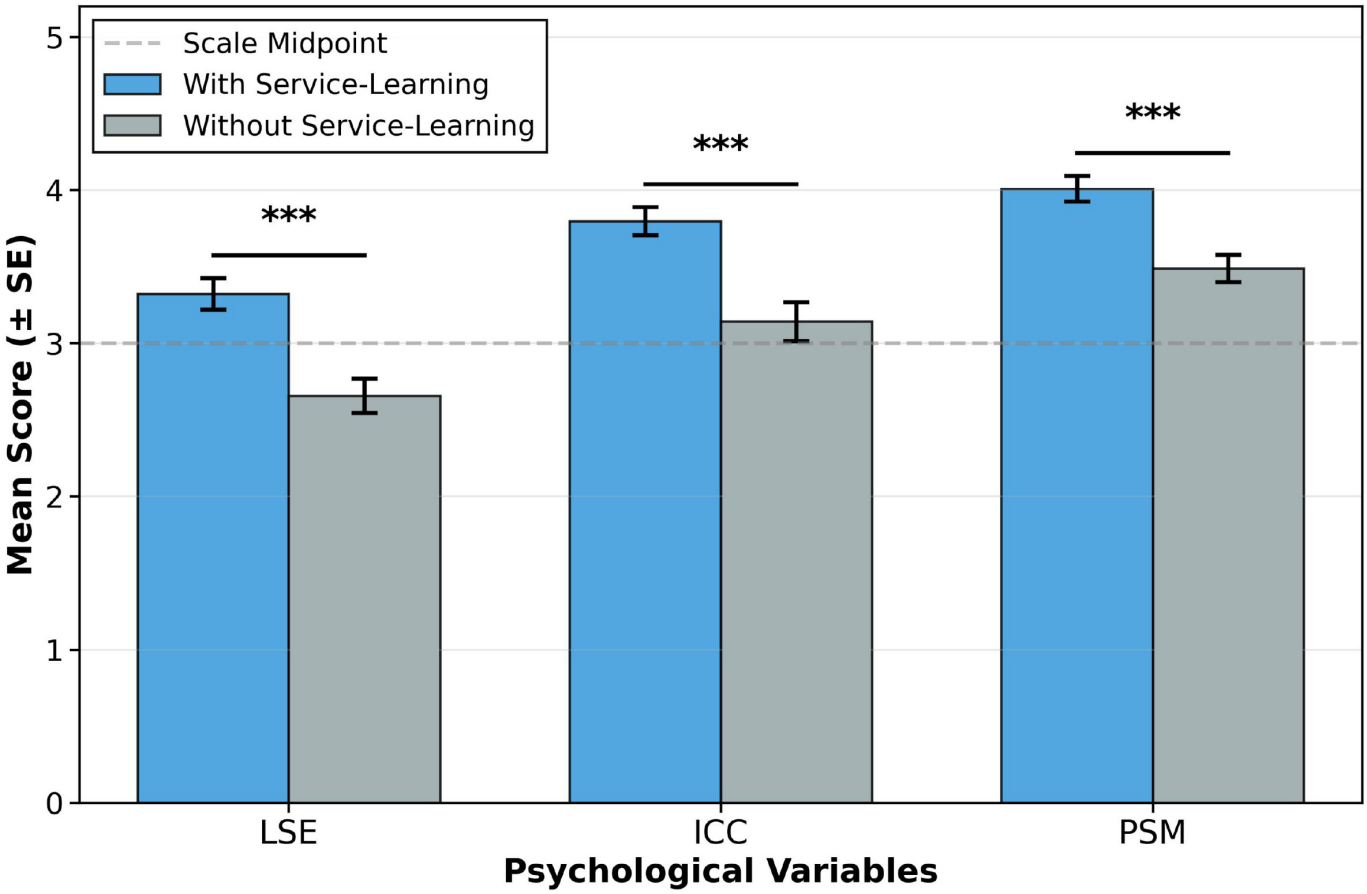
Group differences based on service-learning experience. ***p < 0.001.

Figure 3 vividly illustrates these group differences. Both the statistical and visual results consistently demonstrate that service-learning has a significant positive effect on enhancing students’ core competencies. Therefore, Hypothesis H1 was fully supported.

### 4.3 Regression analysis of core variables

To test the predictive effects of the psychological variables on Public Service Willingness and Perceived Communication Effectiveness (H2a, H2b), two multiple linear regression models were constructed. A multicollinearity test was conducted prior to the analysis, and the Variance Inflation Factor (VIF) for all variables was below 3, well under the critical threshold of 10, indicating no serious multicollinearity issues.

The results of Model 1 show that after controlling for other variables, LSE (β = 0.28, *p* < 0.01), ICC (β = 0.35, *p* < 0.001), and PSM (β = 0.33, *p* < 0.001) all significantly and positively predicted Public Service Willingness, with ICC having the strongest predictive power. Therefore, Hypothesis H2a was supported.

The results of Model 2 show that after controlling for other variables, LSE (β = 0.43, *p* < 0.001) and ICC (β = 0.36, *p* < 0.001) both significantly and positively predicted Perceived Communication Effectiveness, with LSE having the strongest predictive power. Therefore, Hypothesis H2b was supported. The detailed results of these regression models are presented in Table 6.

**TABLE 6:** Regression analysis results for core variables on PSW and PCE.

Model	Independent variable	Unstandardized B	Standardized β	*t*-value	*p*-value
Model 1: Predicting PSW	(Constant)	0.15		0.89	0.375
	LSE	0.25	0.28	3.15	0.002
	ICC	0.38	0.35	4.02	<0.001
	PSM	0.31	0.33	3.88	<0.001
	LA	0.09	0.07	0.91	0.364
	Model Fit: *R*^2^ = 0.62, *F*(4, 103) = 41.25, *p* < 0.001				
Model 2: Predicting PCE	(Constant)	0.21		1.12	0.265
	LSE	0.41	0.43	5.21	<0.001
	ICC	0.33	0.36	4.58	<0.001
	PSM	0.11	0.12	1.35	0.180
	LA	0.05	0.04	0.67	0.504
	Model Fit: *R*^2^ = 0 .58, *F*(4, 103) = 35.18, *p* < 0.001				

Notably, in the regression models, the predictive effect of Language Anxiety (LA) on both dependent variables was not significant.

### 4.4 Mediation effect test of public service motivation

To test the mediating role of Public Service Motivation (PSM) between Language Self-Efficacy (LSE) and Public Service Willingness (PSW) (H3), the PROCESS macro (Model 4) with 5000 bootstrap resamples was used.

The results of the mediation analysis are presented in Table 7 and show the following:

**TABLE 7:** Results of the mediation effect test for PSM (Bootstrap method).

Effect type	Effect	Boot SE	95% CI
Indirect effect (LSE → PSM → PSW)	0.23	0.07	[0.11, 0.38]
Direct effect (LSE → PSW)	0.43	0.09	[0.25, 0.61]
Total effect (LSE → PSW)	0.66	0.08	[0.50, 0.82]

The total effect of LSE on PSW was significant (*c* = 0.66, *p* < 0.001).

Path a: The predictive effect of LSE on the mediator PSM was significant (*a* = 0.51, *p* < 0.001).

Path b and c’: When both LSE and PSM were included in the model to predict PSW, the predictive effect of PSM remained significant (*b* = 0.45, *p* < 0.001), while the direct effect of LSE on PSW (*c’* = 0.43, *p* < 0.001) was still significant but reduced.

The results show that the indirect effect of PSM between LSE and PSW was 0.23, and its 95% bootstrap confidence interval [0.11, 0.38] does not contain 0, indicating a significant mediation effect. The indirect effect accounted for 34.8% of the total effect (0.23/0.66). This suggests that language self-efficacy not only directly influences public service willingness but also indirectly enhances it by boosting students’ public service motivation. Therefore, Hypothesis H3 was supported.

### 4.5 Moderation effect test of language anxiety

To test the moderating role of Language Anxiety (LA) in the relationship between Language Self-Efficacy (LSE) and Perceived Communication Effectiveness (PCE) (H4), the PROCESS macro (Model 1) was used. To avoid multicollinearity, the independent variable (LSE) and the moderator (LA) were centered before creating the interaction term.

**FIGURE 4:**
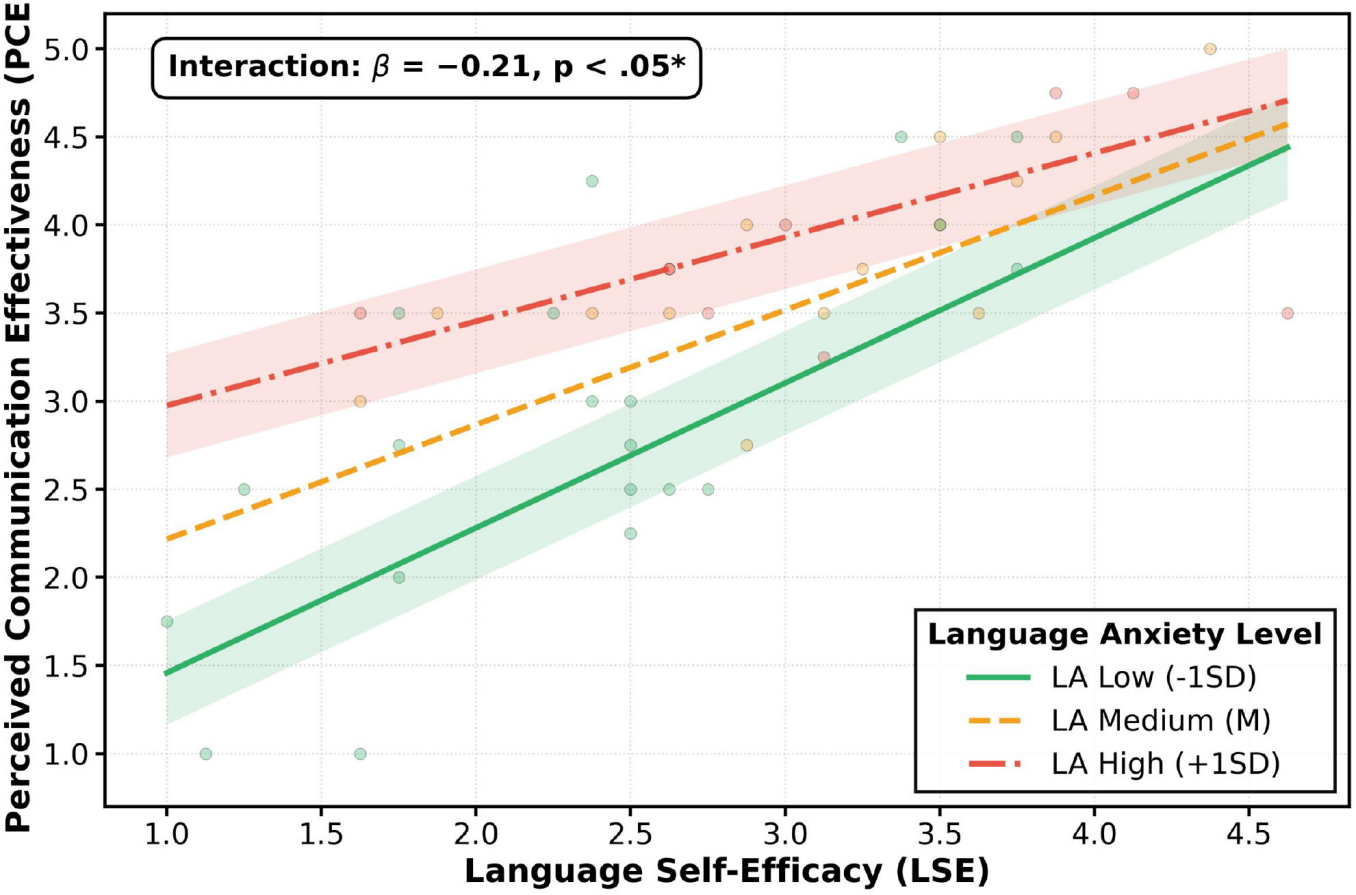
Moderating effect of language anxiety on the LSE-PCE relationship. The figure visualizes the moderating effect. Regression lines depict the LSE-PCE relationship at low (green line; -1 SD), mean (orange line), and high (red line; +1 SD) levels of Language Anxiety. The slope for the low-anxiety group is steep and strong (simple slope = 0.86, *p* < 0.001), while the slope for the high-anxiety group is significantly flatter (simple slope = 0.44, *p* = 0.023). This demonstrates that high anxiety reduces the positive impact of LSE on PCE by 48.8%, illustrating the “system friction” effect. Shaded areas represent 95% confidence intervals.

To visualize the nature of this moderation effect, a simple slope analysis was conducted and plotted in Figure 4. As shown, at high levels of language anxiety, the positive impact of language self-efficacy on perceived communication effectiveness was significantly weakened. Therefore, Hypothesis H4 was supported. The detailed results of the moderation model are presented in Table 8.

**TABLE 8:** Results of the moderation effect test for LA.

Model variable	*B*	SE	*t*-value	*p*-value
(Constant)	3.41	0.08	42.15	<0.001
LSE (centered)	0.65	0.09	7.22	<0.001
LA (centered)	0.52	0.11	4.73	<0.001
LSE × LA (Interaction)	–0.21	0.08	–2.63	0.010

The interaction term between LSE and LA was significant (*B* = -0.21, *p* = 0.010), confirming that Language Anxiety negatively moderates the relationship between LSE and PCE. Simple slope analysis, visualized in Figure 4, confirmed that the effect of LSE on PCE was significant at both low (-1 SD) and high (+1 SD) levels of Language Anxiety, but the effect was significantly stronger in the low-anxiety condition.

Through a series of progressive statistical analyses, the research hypotheses were systematically tested. The results indicate that service-learning experience significantly enhances students’ core psychological competencies. LSE, ICC, and PSM are key predictors of public service willingness and communication effectiveness. PSM plays an important “bridging” role between confidence and willingness. Meanwhile, language anxiety acts as a “moderator,” inhibiting the conversion of confidence into effective communication at high levels.

## 5 Discussion

The empirical results presented in Chapter 4 are not merely statistical artifacts; they suggest the operational dynamics of a complex psychological system. A thematic analysis of these findings allows for a deeper theoretical dialogue, challenging simplistic notions of competence and revealing the intricate mechanisms that govern how latent potential is converted into tangible public service performance.

### 5.1 Redefining “competence”—from static resources to a dynamic system

Traditional approaches in language education often treat competence as an inventory of static resources (e.g., linguistic knowledge, cultural facts). Our findings compel a reconceptualization toward a dynamic system model. The regression results (Table 5) demonstrate that even high levels of Intercultural Communicative Competence (ICC), a key resource, do not guarantee high Public Service Willingness (PSW) or Perceived Communication Effectiveness (PCE) in isolation. System performance appears to be an emergent property arising from the interplay between ICC, Language Self-Efficacy (LSE), and Public Service Motivation (PSM). Furthermore, the low mean score of LSE (*M* = 2.92) amidst high motivation (PSM *M* = 3.62) highlights a critical insight: a system rich in motivation but poor in self-perceived capability is fundamentally imbalanced and prone to underperformance. This suggests that educational optimization should shift from merely “filling” students with resources to “calibrating” the dynamic relationships between these internal system states.

### 5.2 The affective hub of motivation—igniting the conversion of confidence into action

A central finding of this study is the powerful mediating role of Public Service Motivation (PSM), which functions as the affective hub of the system. The mediation analysis (Table 6) shows that a significant portion (34.8%) of the effect of LSE on PSW is channeled through PSM. This mechanism provides a crucial counterpoint to purely cognitive models of language application. It suggests that confidence (LSE), a cognitive belief, remains inert potential until it is “ignited” by an affective, value-driven engine (PSM). In systemic terms, PSM acts as a catalyst or amplifier, converting the potential energy of self-efficacy into the kinetic energy of behavioral willingness. This finding enriches the work of scholars like Hu et al. (2025) and Tang et al. (2024) by empirically mapping the precise pathway through which intrinsic motivation translates cognitive states into pro-social intentions within a specific professional context, thus providing a more granular understanding of PSM’s function.

### 5.3 The non-linear paradox of anxiety—the phenomenon of “system friction” in high-capability individuals

A counterintuitive finding emerges from the moderation analysis: language anxiety was significantly correlated with all key variables (Table 4), yet its direct regression effect on PCE was non-significant (Table 6). This apparent contradiction is resolved when considering its role as a boundary condition rather than a direct driver. The significant interaction term (Table 8, β = -0.21, *p* = 0.010) demonstrates that LA operates non-linearly, selectively suppressing the conversion efficiency of LSE into PCE only at elevated anxiety levels. This finding aligns with the concept of “system friction” and provides empirical detail to the theoretical arguments of Ma (2022) by showing *how* anxiety undermines performance—not by being a direct negative predictor, but by acting as a constraint on the positive influence of self-efficacy. It also echoes the Yerkes-Dodson law, suggesting optimal arousal zones for performance. For educational practice, this finding underscores that anxiety management interventions should be stratified—targeting high-anxiety subgroups rather than universal application.

## 6 Conclusion

This study, through rigorous quantitative methods, illuminates the psychological architecture associated with English majors’ commitment to public service. It confirms that the system’s outputs—Public Service Willingness and Communication Effectiveness—emerge not as a simple sum of individual traits, but from the dynamic interplay of core psychological competencies.

The empirical model identifies Language Self-Efficacy (LSE), Intercultural Communicative Competence (ICC), and Public Service Motivation (PSM) as pivotal psychological control points. Furthermore, the research validates a critical transformation pathway wherein LSE is associated with maximum service willingness. Perhaps most critically, the study identifies and quantifies Language Anxiety (LA) as a key System Boundary Condition, revealing its non-linear friction effect on the conversion of confidence into communicative effectiveness.

Consequently, this study provides an empirical foundation for a paradigm shift in foreign language education—from a focus on accumulating discrete skills to one of systemic optimization. By strategically enhancing the identified control points, addressing the primary bottleneck (LSE), and mitigating the boundary constraints imposed by LA, educational institutions can more systematically cultivate the high-caliber linguistic talent required to meet pressing national and societal needs.

### 6.1 Limitations and future directions

While this research offers a robust model of the psychological dynamics in foreign language talent cultivation, its inherent limitations also chart a course for future inquiry.

First, the sample size (*N* = 108), while demonstrated to be adequate for the regression and PROCESS macro analyses conducted, remains modest. While the sample was drawn from multiple universities within a single Chinese province enhances regional applicability, the findings, the results may not be fully generalizable to other cultural or national contexts. The educational systems and sociocultural factors influencing language learners can vary significantly across different countries (Gkintoni et al., 2025). Therefore, future research should seek to replicate this study with larger, more diverse samples in diverse international settings to ascertain the model’s cross-cultural robustness and identify potential context-specific boundary conditions.

Second, the cross-sectional design offers a static snapshot of the relationships between variables. Although our mediation and moderation analyses suggest directional pathways consistent with the proposed theoretical framework, they cannot establish definitive causality. The language used throughout this paper has been carefully tempered to reflect this statistical, rather than causal, basis. It is plausible, for instance, that a reciprocal relationship exists where higher PSM motivates students to engage in activities that, in turn, bolster their LSE. Longitudinal research that tracks students throughout their academic careers is essential for establishing the causal direction and dynamic evolution of these psychological variables.

Third, although the study’s measurement model was validated through Confirmatory Factor Analysis and common method bias was found not to be a substantial threat via Harman’s single-factor test, the reliance on self-report data at a single time point means the influence of social desirability bias cannot be entirely ruled out. To strengthen the objectivity of the conclusions, future studies should incorporate multi-source data and more objective metrics, such as behavioral observations during simulated public service tasks or third-party performance-based assessments of communication effectiveness to triangulate the findings.

Building on these limitations, future research could advance in several promising directions: (1) Targeted Intervention Studies: Designing and testing specific pedagogical interventions (e.g., workshops focused on building LSE or seminars encouraging reflection on PSM) to empirically validate their impact on the system’s control points. (2) In-depth Qualitative Inquiry: Conducting interviews or case studies to explore the lived experiences of students, which would be particularly valuable for gaining a more nuanced understanding of how high language anxiety functions as “system friction” for otherwise highly efficacious individuals.

## Data Availability

The original contributions presented in the study are included in the article/Supplementary material, further inquiries can be directed to the corresponding author.

## References

[B1] Bru-Luna L. M. Martí-Vilar M. Merino-Soto C. Cervera-Santiago J. L. (2021). Emotional intelligence measures: A systematic review. *Healthcare* 9:1696. 10.3390/healthcare9121696 34946422 PMC8701889

[B2] Bandura A. (1997). *Sources of self-efficacy. Self-efficacy: The exercise of control.* New York, NY: W H Freeman, 79–113.

[B3] Byram M. (1997). *Teaching and assessing intercultural communicative competence.* Bristol: Multilingual Matters.

[B4] Chan C. K. Y. (2023). A comprehensive AI policy education framework for university teaching and learning. *Int. J. Educ. Technol. High. Educ.* 20:38. 10.1186/s41239-023-00408-3

[B5] Chen T. Sun S. (2025). Evaluating automated evaluation systems for spoken English proficiency: An exploratory comparative study with human raters. *PLoS One* 20:e0320811. 10.1371/journal.pone.0320811 40153336 PMC11952242

[B6] Clark L. A. Watson D. (2019). Constructing validity: New developments in creating objective measuring instruments. *Psychol. Assess.* 31 1412–1427. 10.1037/pas0000626 30896212 PMC6754793

[B7] Conti G. (2025). *‘You shall know a word by the company it keeps’ – Why and how this sentence revolutionised my teaching 25 years ago. The language gym.* Available online at: https://gianfrancoconti.com/2025/03/ (accessed April 15, 2025).

[B8] Dankwa-Mullan I. (2024). Health equity and ethical considerations in using artificial intelligence in public health and medicine. *Prevent. Chronic Dis.* 21:240245. 10.5888/pcd21.240245 39173183 PMC11364282

[B9] Dorninger C. Abson D. J. Apetrei C. I. Derwort P. Ives C. D. Klaniecki K. (2020). Leverage points for sustainability transformation: A review on interventions in food and energy systems. *Ecol. Econ.* 171:106570. 10.1016/j.ecolecon.2019.106570

[B10] East M. Tolosa C. Howard J. Biebricher C. Scott A. (2022). *Beginning the journeys towards intercultural capability. In journeys towards intercultural capability in language classrooms.* Berlin: Springer, 1–23. 10.1007/978-981-19-0991-7_1

[B11] Emir G. Yangın-Ekşi G. (2024). The role of telecollaboration in English language teacher education: A systematic review. *Smart Learn. Environ.* 11:3. 10.1186/s40561-024-00290-0

[B12] Fan L. Cui F. (2024). Mindfulness, self-efficacy, and self-regulation as predictors of psychological well-being in EFL learners. *Front. Psychol.* 15:1332002. 10.3389/fpsyg.2024.1332002 38601825 PMC11004504

[B13] Gkintoni E. Antonopoulou H. Sortwell A. Halkiopoulos C. (2025). Challenging cognitive load theory: The role of educational neuroscience and artificial intelligence in redefining learning efficacy. *Brain Sci.* 15:203. 10.3390/brainsci15020203 40002535 PMC11852728

[B14] Gutiérrez-Santiuste E. Ritacco-Real M. (2023). Intercultural communicative competence in higher education through telecollaboration: Typology and development. *Educ. Information Technol.* 28 4887–4914. 10.1007/s10639-023-11751-3 37361747 PMC10067007

[B15] Hackett S. Janssen J. Beach P. Perreault M. Beelen J. van Tartwijk J. (2023). The effectiveness of Collaborative online international learning (COIL) on intercultural competence development in higher education. *Int. J. Educ. Technol. High. Educ.* 20:5. 10.1186/s41239-022-00373-3 36713634 PMC9870663

[B16] Hayes A. F. (2022). *Introduction to mediation, moderation, and conditional process analysis: A regression-based approach*, 3rd Edn. New York, NY: The Guilford Press.

[B17] Horwitz E. K. Horwitz M. B. Cope J. (1986). Foreign language classroom anxiety. *Modern Lang. J.* 70 125–132. 10.2307/327317

[B18] Hu Z. X. Gan K. P. Sun G. Y. Wang Q. (2025). Public service motivation and career choice intentions of social work students: The roles of altruistic motivation and professional values. *Front. Psychol.* 16:1517457. 10.3389/fpsyg.2025.1517457 40083759 PMC11903731

[B19] Jacobson M. J. Levin J. A. Kapur M. (2019). Education as a complex system: Conceptual and methodological implications. *Educ. Res.* 48 112–119. 10.3102/0013189X19826958 38293548

[B20] Lee T. Y. Ho Y. C. Chen C. H. (2023). Integrating intercultural communicative competence into an online EFL classroom: An empirical study of a secondary school in Thailand. *Asian Pac. J. Sec. For. Lang. Educ.* 8:4. 10.1186/s40862-022-00174-1

[B21] Li L. (2022). Reskilling and upskilling the future-ready workforce for industry 4.0 and beyond. *Inf. Syst. Front.* 24 1541–1556. 10.1007/s10796-022-10308-y 35855776 PMC9278314

[B22] Linnér B. O. Wibeck V. (2021). Drivers of sustainability transformations: Leverage points, contexts and conjunctures. *Sustainability Sci.* 16 889–900. 10.1007/s11625-021-00957-4 33936316 PMC8075710

[B23] Luo Q. Ahmadi R. Izadpanah S. (2024). Exploring the mediating role of self-efficacy beliefs among EFL university language learners: The relationship of social support with academic enthusiasm and academic vitality. *Heliyon* 10:e33253. 10.1016/j.heliyon.2024.e33253 39022045 PMC11252873

[B24] Ma Y. (2022). The triarchy of L2 learners’ emotion, cognition, and language performance: Anxiety, self-efficacy, and speaking skill in lights of the emerging theories in SLA. *Front. Psychol.* 13:1002492. 10.3389/fpsyg.2022.1002492 36204743 PMC9530130

[B25] Perry J. L. (1996). Measuring public service motivation: An assessment of construct reliability and validity. *J. Public Adm. Res. Theory* 6 5–22. 10.1093/oxfordjournals.jpart.a024303

[B26] Qin X. (2024). Collaborative inquiry in action: A case study of lesson study for intercultural education. *Asian Pac. J. Sec. For. Lang. Educ.* 9:66. 10.1186/s40862-024-00294-w

[B27] Schauer G. A. (2024). *Intercultural competence and pragmatics.* Berlin: Springer Nature, 10.1007/978-3-031-44472-2

[B28] Selin N. E. Giang A. Clark W. C. (2023). Progress in modeling dynamic systems for sustainable development. *Proc Natl. Acad. Sci.* 120:e2216656120. 10.1073/pnas.2216656120 37751553 PMC10556647

[B29] Shadiev R. Yu J. Sintawati W. (2021). Exploring the impact of learning activities supported by 360-degree video technology on language learning, intercultural communicative competence development, and knowledge sharing. *Front. Psychol.* 12:766924. 10.3389/fpsyg.2021.766924 34899512 PMC8663917

[B30] Tang H. An S. Zhang L. Xiao Y. Li X. (2024). The antecedents and outcomes of public service motivation: A meta-analysis using the job demands–resources model. *Behav. Sci.* 14:861. 10.3390/bs14100861 39457733 PMC11505570

[B31] Tran T. Q. Duong T. M. (2018). The effectiveness of the intercultural language communicative teaching model for EFL learners. *Asian Pac. J Sec. Foreign Lang. Educ.* 3:6. 10.1186/s40862-018-0048-0

[B32] Wibowo A. H. Mohamad B. Djatmika Santosa R. (2024). Designing and assessing experiential learning pedagogy for an intercultural communicative competence training module: A quasi-experimental study. *Front. Educ.* 9:1470209. 10.3389/feduc.2024.1470209

[B33] Zhang Q. Mohammad Ismail M. I. R. Bin Zakaria A. R. (2025). Enhancing intercultural competence in technical higher education through AI-driven frameworks. *Sci. Rep.* 15:22019. 10.1038/s41598-025-03303-1 40594064 PMC12217878

[B34] Zhou R. Samad A. Perinpasingam T. (2024). A systematic review of cross-cultural communicative competence in EFL teaching: Insights from China. *Humanities Soc. Sci. Commun.* 11:1750. 10.1038/s41599-024-04071-5

