# Supplementary material for: Boundary conditions for foreign language talent public service output
Source: Front Psychol. 2026 Jan 26;17:1748812. doi: 10.3389/fpsyg.2026.1748812 (PMC12883816; doi:10.3389/fpsyg.2026.1748812)
Supplement: Supplementary file 1 [file Data_Sheet_1.pdf]

System Optimization in Education: Identifying Critical Psychological Control Points and System Boundary Conditions for Foreign Language Talent Public Service Output

Dear Student:

Thank you for participating in our academic study aimed at modeling the psychological system that enables English majors to excel in public service. This survey is designed to gather quantitative data on the key psychological variables—such as confidence, motivation, and intercultural skills—that function as critical components within this system.

Your responses are crucial to this research. This survey is anonymous, and all data will be used for academic purposes only. Your personal information will be kept strictly confidential. The survey will take approximately 10-15 minutes to complete.

Thank you for your time and participation!

Part I: Background Information & Public Service Experience

Please fill in or select the option that best applies to you.

1. Your Gender:

A. Male

B. Female

C. Prefer not to say

2. Your Academic Year:

A. Freshman

B. Sophomore

C. Junior

D. Senior

E. Graduate Student

F. Other (Please specify: __________)

3. Your Academic Concentration (within the English major, multiple selections allowed):

A. English Language and Literature

B. Business English

C. Translation and Interpreting

D. English Education

E. Other (Please specify: __________)

4. How would you rate your overall English proficiency?

A. Basic

B. Intermediate

C. Good

D. Fluent

E. Highly Fluent / Near-Native

5. Have you participated in any public service activities (e.g., volunteer translation, cultural exchange events, community service, international conference support, services for international patients/tourists, etc.)?

A. Yes (Please continue to questions 6-7)

B. No (Please skip to question 8)

6. If yes, what types of public service have you primarily been involved in? (Multiple selections allowed)

A. Cultural Exchange / Tour Guiding

B. Volunteer Translation / Interpretation

C. International Community Service

D. International Conference / Event Support

E. Health-related Public Service for international visitors

F. Volunteer Teaching / Language Instruction

G. Other (Please specify: __________)

7. If you have public service experience, how often do you participate?

A. Never

B. Occasionally

C. 1-2 times a year

D. 1-2 times a month

E. Once a week or more

8. Does your English major curriculum include a Service-Learning program (i.e., a course that integrates academic study with community service)?

A. Yes

B. No

C. Not sure

Part II: Linguistic Self-Efficacy

Based on your personal feelings and past experiences, please rate your level of agreement with the following statements.

(1=Strongly Disagree, 2=Disagree, 3=Neutral, 4=Agree, 5=Strongly Agree)

No.	Statement	1	2	3	4	5
1	I am confident in my ability to introduce Chinese culture or local features to foreigners in English.					
2	I can confidently handle unexpected situations or unclear information in a public service setting using English.					
3	Even when I encounter unfamiliar words or complex phrases, I am confident I can communicate effectively in English during public service.					
4	I believe I can clearly and accurately explain complex information related to public service in English.					
5	I am confident in my ability to understand the accents or non-standard pronunciations of international service recipients.					
6	I can confidently interact with people from different cultural backgrounds in a public service setting using English.					
7	I believe I can overcome any English communication barriers I encounter in public service.					
8	I am confident in my ability to express my views and persuade others in English.					

Part III: Intercultural Communicative Competence

Based on your personal feelings and past experiences, please rate your level of agreement with the following statements.

(1=Strongly Disagree, 2=Disagree, 3=Neutral, 4=Agree, 5=Strongly Agree)

No.	Statement	1	2	3	4	5
9	I am aware of the cultural differences in how people from various countries communicate in public settings.					
10	I can understand and accept the perspectives and behaviors of people from cultures different from my own.					
11	In intercultural communication, I can flexibly adjust my verbal and non-verbal behaviors to adapt to the other person.					
12	I can effectively manage misunderstandings or conflicts that arise during intercultural communication.					
13	I am eager to learn about and understand the values and lifestyles of different cultures.					
14	When communicating with people from other countries, I am sensitive to potential cultural differences.					
15	I am able to build positive relationships with people from diverse cultural backgrounds.					
16	When faced with intercultural differences, I typically maintain an open and respectful attitude.					

Part IV: Public Service Motivation

Based on your personal feelings, please rate your level of agreement with the following statements.

(1=Strongly Disagree, 2=Disagree, 3=Neutral, 4=Agree, 5=Strongly Agree)

No.	Statement	1	2	3	4	5
17	I want to use my skills as an English major to contribute to the public good.					
18	Helping others brings me a sense of satisfaction.					
19	I am willing to dedicate my time and energy to public service.					
20	Serving the public is important to me.					
21	I am eager to get involved in activities that address social issues.					
22	I feel it is my responsibility to serve the community.					

No.	Statement	1	2	3	4	5
23	I enjoy helping people in need.					

Part V: Language Anxiety in Public Service Contexts

Based on your personal feelings and past experiences, please rate how frequently you experience the following.

(1=Never, 2=Rarely, 3=Sometimes, 4=Often, 5=Always)

No.	Statement	1	2	3	4	5
24	I feel nervous when communicating with foreigners in English during public service.					
25	I worry that making mistakes in English during public service will negatively affect the quality of the service.					
26	When communicating in English for public service, I sometimes feel overwhelmed and unsure of how to express myself.					
27	Even when I am well-prepared, I still feel anxious when speaking English in a public service role.					
28	When I express myself in English during public service, I am afraid of being misunderstood.					
29	I tend to avoid initiating conversations in English with foreigners in public service settings.					

Part VI: Willingness for Public Service & Perceived Communication Effectiveness

Based on your personal feelings and past experiences, please rate your level of agreement with the following statements.

(1=Strongly Disagree, 2=Disagree, 3=Neutral, 4=Agree, 5=Strongly Agree)

No.	Statement	1	2	3	4	5
Willingness for Public Service						
30	I am very willing to use my English skills to participate in more public service activities.					
31	If given the opportunity in the future, I will proactively engage in public service involving international communities.					

No.	Statement	1	2	3	4	5
32	I believe it is very meaningful to use my English proficiency to serve society.					
Perceived Communication Effectiveness						
33	I believe my English communication in public service is clear and effective.					
34	In public service, I can accurately understand the needs of service recipients and respond effectively in English.					
35	Service recipients generally understand the information I convey in English.					
36	I believe my English communication skills play a positive role in solving problems in public service contexts.					

End of Survey

Thank you again for taking the time to complete this questionnaire!
